# Antimicrobial Effects of Chitosan, Mastic Essential Oil and Citric Acid, and Their Combinations on the Spoilage Microbiota of “Guacamole”: An Avocado-Based Salad

**DOI:** 10.3390/foods14213796

**Published:** 2025-11-06

**Authors:** Rameez Al-Daour, Ayman Zehra Shakir Hussein, Tareq M. Osaili, Fayeza Hasan, Dinesh Kumar Dhanasekaran, Mutamed Ayyash, Leila Cheikh Ismail, Thaís Benincá, Patrícia da Silva Malheiros, Ioannis N. Savvaidis

**Affiliations:** 1 Department of Environmental Health Sciences, College of Health Sciences, University of Sharjah, Sharjah 27272, United Arab Emirates; rameezdaour@hotmail.com; 2 Department of Clinical Nutrition and Dietetics, College of Health Sciences, University of Sharjah, Sharjah 27272, United Arab Emirates; aymanzshakir@gmail.com (A.Z.S.H.); tosaili@sharjah.ac.ae (T.M.O.);; 3 Research Institute of Medical and Health Sciences (RIMHS), University of Sharjah, Sharjah 27272, United Arab Emirates; u00040620@sharjah.ac.ae (F.H.); ddhanasekaran@sharjah.ac.ae (D.K.D.); 4 Department of Nutrition and Food Technology, Faculty of Agriculture, Jordan University of Science and Technology, Irbid 22110, Jordan; 5 Department of Food, Nutrition and Health, United Arab Emirates University, Al Ain 15551, United Arab Emirates; mutamed.ayyash@uaeu.ac.ae; 6 Nuffield Department of Women’s & Reproductive Health, University of Oxford, Oxford OX1 2JD, UK; 7 Microbiology and Food Hygiene Laboratory, Food Science and Technology Institute, Federal University of Rio Grande do Sul, Porto Alegre 90010-150, Rio Grande do Sul, Brazil; thais_beninca@hotmail.com; 8 Laboratory of Food Chemistry and Food Microbiology, Department of Chemistry, University of Ioannina, 45110 Ioannina, Greece

**Keywords:** avocado, guacamole, natural antimicrobials, microbial spoilage, essential oils, ready-to-eat

## Abstract

Guacamole is an avocado-based Ready-to-Eat (RTE) salad product, consumed globally and increasingly popular due to health trends. Its composition, characterized by a relatively neutral pH and stable water activity, creates favorable conditions for microbial proliferation, leading to spoilage, and resulting in a limited shelf-life of the product. Natural antimicrobials, such as essential oils (EOs), organic acids, chitosan, etc. have the potential to control microbial populations, therefore delaying spoilage and consequently providing a shelf-life extension. This study evaluated the effects of different concentrations of the selected natural antimicrobials [citric acid, mastic essential oil (EO), chitosan] added, either singly or combined, on the indigenous microbial (“spoilage”) association of guacamole during two storage conditions (chill, mild) for a period of 7 days. Results showed that of all the species enumerated in the present study, yeasts and molds were the predominant species, followed by Lactic Acid Bacteria (LAB), *Enterobacteriaceae* and *Pseudomonas* spp., at populations ranging from 3.6 to 2.0 and 2.65 to 1.45 log CFU/g, at 4 and 10 °C, respectively, in the control (CNL) avocado-based salad samples. Reductions in the range 1 to 2 log CFU/g were obtained for *Pseudomonas* spp., *Enterobacteriaceae*, and yeasts and molds under the triple antimicrobial treatment (citric acid, mastic EO, chitosan; CACHM), whereas interestingly, for LAB the highest reduction (1.74 log CFU/g) was achieved by chitosan and mastic EO (CHM), followed by CACHM (1.5 log CFU/g). Refrigeration (chill, 4 °C) as a hurdle acted as an additional barrier delaying microbial growth in all samples. To our knowledge, this is the first study to (a) evaluate the effect of natural antimicrobials (added, either singly or combined), namely citric acid, mastic EO, and chitosan on the microbiota of guacamole and (b) assess the possible application of the aforementioned natural antimicrobials in potentially increasing the shelf-life of a RTE avocado-based product (guacamole, in this study).

## 1. Introduction

Guacamole is a widely consumed salad dip or spread, primarily prepared basically with fresh avocado (mashed), frequently combined with fresh chopped onion, cilantro, and tomato [1]. Guacamole is nowadays available as a prepackaged, refrigerated (4 °C) RTE avocado salad dip in retails stores, or it may also be prepared as a fresh salad at home. Consumers’ increasing awareness for healthy eating of plant-based products (including avocado, as shown on digital platforms) has driven high demand for avocado-based products (guacamole, salsa, etc.) [2]. In 2016, the import value of guacamole in the United States was approximately 218 million dollars [3]. Interestingly, in 2024, fresh avocado imports reached a record value of 3.8 billion dollars [4].

With the growing popularity for various avocado-based products, such as guacamole, salsa, etc., concerns about the microbial quality and safety of such RTE products involving no additional processing (i.e., heat treatment) by the consumers have been raised [5]. The pH of guacamole (4.8), its high-water activity (a_w_ ≈ 0.99), and unsaturated lipid content provide conditions ideal for microbial proliferation, growth, and subsequent spoilage of the avocado-based products [6]. A study has identified elevated numbers of aerobic (mesophilic) bacteria, coliforms, *Staphylococcus aureus*, and yeasts, and molds in avocados as the main microbial consortia of guacamole [7]. Additionally, in another study, spoilage microorganisms (psychrotrophic bacteria) were also enumerated with counts ranging from <2.0 to 9.44 log CFU/g, as well as yeasts and fungi, ranging from <1.0 to 6.67 log CFU/g [8].

Food products’ contamination, along the production chain, coupled at times with inadequate processing and poor storage (i.e., temperature abuse), all are contributing factors to microbial proliferation, potentially reducing shelf-life [9]. This is particularly relevant for RTE foods, including salad formulations such as guacamole, posing potential food safety hazards (for the immunocompromised, pregnant women, etc.). To extend shelf-life and safeguard the microbial safety of perishable products, especially for export purposes, effective antimicrobial strategies/interventions are therefore needed that avoid adverse effects on the product’s sensory profile and allow it to be economically viable [10].

Chitosan, a natural polymer extracted from chitin, found in the shells of crustaceans such as crabs, shrimp, and crawfish [11], has demonstrated antimicrobial activity through multiple mechanisms, including inhibition of DNA transcription, disruption of cell membrane lipids [12], modulation of cell permeability [13], and impairment or degradation of microbial cell walls [14,15]. Chitosan has an acidic type of flavor (“lemony”), and in some cases, could negatively affect product flavor (depending on the amount added) and decrease palatability. Therefore, combining chitosan with other additives (i.e., EOs) could offer a promising strategy to maintain its antimicrobial efficacy while minimizing any negative sensory impact in minimally processed RTE foods, such as guacamole [10].

Citric acid (having a low pH) is widely used in the food industry, particularly in fruit-based products, beverages, and juices, due to its preservative properties. In its salt form (i.e., potassium citrate) it is a widely employed preservative and is Generally Regarded as Safe (GRAS), contributing to microbial stability and control, primarily through pH reduction and chelation of essential minerals interfering with cells microbial metabolism. Moreover, the use of citric acid prevents enzymatic browning, stabilizing flavor and color of products, making it a suitable additive in minimally processed foods [16].

Mastic gum, a natural resin extracted from the *Pistacia lentiscus* tree, and especially its EO (obtained usually by hydro-distillation), has shown significant antimicrobial properties against various microorganisms [17,18] due to the presence of bioactive compounds, such as α-pinene, β-myrcene, β-pinene, limonene, β-caryophyllene, verbenone, α-terpineol, and linalool. These compounds may act synergistically, exerting antibacterial activity against both Gram-positive and Gram-negative bacteria [19].

To date, and to our present knowledge, no study has evaluated the effects (single or combined) of chitosan (CH, 0.5–1% w/w), citric acid (CA, 0.15–0.3% w/w), and mastic EO (M, 0.2–0.4% w/w) on the microbiota (“spoilage”) organisms in guacamole, stored at 4 or 10 °C for 7 days. Therefore, the aim of our study was to investigate the behavior of evolution of the microbiota (spoilage-causing microorganisms) during storage of the guacamole under the aforementioned treatments. The present study was primarily designed to establish the evolution of the microbiota, both in the absence and presence of various natural antimicrobials of guacamole without, however, establishing either the sensorial or microbiological shelf-life.

## 2. Materials and Methods

### 2.1. Preparation of Guacamole

Fresh avocados (“Huss” variety), cilantros, tomatoes, onions, jalapeños, and salt (table) were purchased from a local (Sharjah, UAE) supermarket. Avocados (raw, unpeeled) were covered under a plastic sheet membrane film and allowed to mature at room temperature (24 °C) for 2 days. The guacamole was prepared by mixing (in the following sequence): Mashed avocados (71% w/w), diced tomatoes (10% w/w), diced onions (8% w/w), freshly squeezed lemon juice (5% w/w), diced jalapeño peppers (3.5% w/w), chopped cilantros (1.5% w/w) and salt (1% w/w). Kitchen utensils, lemon squeezers, spoons, knives, cutting boards, and salad containers were, all prior to use, sterilized using an alcohol 70% v/v solution.

### 2.2. Preparation of Antimicrobial Solutions

A chitosan (CH) solution was prepared using low-molecular weight chitosan (50–160 kDa) with a deacetylation degree of 75–85% (Sigma-Aldrich, St. Louis, MO, USA). Water diluted and mixed in a 0.33% (v/v) acetic acid solution resulted in the preparation of a chitosan emulsion solution, yielding finally two concentrations of chitosan (0.5% and 1% w/w). Mastic (M), pure organic EO, was shipped from a retailer based abroad (Mastihashop, Chios, Greece). Two concentrations (0.2% and 0.4% v/v) of mastic EO were prepared. Additionally, potassium citrate (a salt of citric acid) acquired from a local company was prepared as a citric acid (CA) solution to be used at concentrations of 0.15% and 0.3% (w/v). Mixed (two or three) solutions, involving the aforementioned single antimicrobials, were always freshly prepared and mixed to the desirable final concentrations (described below). Concentrations of natural antimicrobials, finally added to the guacamole, were previously determined from preliminary trial experiments, establishing a minimum antimicrobial effect on the aerobic mesophilic bacteria of the guacamole (a 1-day study) without at the same time negatively affecting the sensorial quality of the guacamole samples.

### 2.3. Addition of Antimicrobials

The guacamole (untreated or treated) samples were divided into a total of eleven groups, including the following: Untreated CNL—control (absence of antimicrobials) and treated CA—citric acid 0.15%; CA—citric acid 0.3%; M—mastic EO 0.2%; M—mastic EO 0.4%; CH—chitosan 0.5%; CH—chitosan 1%; CAM—citric acid 0.3% + mastic EO 0.4%; CACH—citric acid 0.3% + chitosan 1%; CHM—chitosan 1% + mastic EO 0.4%; and CACHM—citric acid 0.3% + chitosan 1% + mastic EO 0.4%. All solution concentrations are expressed in v/v.

All preparations (including microbiological enumerations) were carried inside a Class II Biosafety Cabinet (5’ Purifier Logic+, Labconco, Kansas City, MO, USA) to ensure a clean and germ-free environment. The guacamole samples were then individually placed in sterile, airtight containers, stored at two different temperatures, 4 and 10 °C, in cooling incubators set at these temperatures. Samples (control and treated) were withdrawn on days 0, 1, 3, 5, and 7 to evaluate the evolution of the microbiota of the guacamole samples.

### 2.4. Microbial Enumeration

After the storage period, samples were aseptically transferred to stomacher bags containing 90 mL of peptone water (Himedia, Mumbai, India) and homogenized for 60 s using a stomacher (Interscience; Saint Nom la Brétèche, France). Appropriate decimal dilutions were prepared in sterile buffered peptone water (10% v/v) and 1 mL of each serial dilution was poured in or plated on dry media plates, and in duplicate, using the either the pour or spread method. For the enumeration of the Total Plate Count (TPC; aerobic mesophilic bacteria), Place Count Agar (PCA, Himedia, Mumbai, India) was used, and plates were incubated at 35 °C for 2 days. Yeasts, and molds (Y&M) were counted using Sabouraud Dextrose Agar (SDA, Himedia, Mumbai, India) and plates were incubated at 25 °C for 5 days. LAB were enumerated on De Man, Rogosa and Sharpe (MRS, Himedia, Mumbai, India) Agar using the pour plate method, under appropriate incubation conditions (35 °C, 3–4 days). *Enterobacteriaceae* were enumerated on Violet Red Bile Glucose Agar (VRBGA, Himedia, Mumbai, India), and plates incubated at 35 °C for 1 day. *Pseudomonads* spp. were enumerated using the spread method on *Pseudomonas* Agar, supplemented with Cetrimide–Fucidin–Cephaloridine (CFC) supplement, and plates aerobically incubated at 25 °C for 2 days. Colonies, on average 25–250 on Agar solid media plates, were manually determined using a Stuart^®^ Colony Counter (Cole-Parmer, Eaton Socon, UK). Results were converted as log values and expressed as log Colony Forming Unit (CFU)/g.

### 2.5. Statistical Analysis

A total quantity of approximately 1110 g of freshly mashed prepared guacamole (involving 10 g samples; 11 treatments; five sampling days; and two temperatures) was used. Three individually conducted (separate experiments/replicates) were conducted within a period of approximately 6 weeks. Statistical data analysis was conducted using IBM SPSS Statistics software (version 26, Chicago, IL, USA). First, a two-way analysis of variance (ANOVA) was performed. Then, a post hoc analysis was conducted using Tukey’s honestly significant difference (HSD) test. These statistical tests assessed the impact of antimicrobial treatments and storage duration on the viability of the tested microorganisms, measured in log CFU/g, as well as their potential interactions. A significance level of *p* < 0.05 was considered statistically (significant) for all microbiological analyses.

## 3. Results and Discussion

The indigenous microflora related a food product, plus microorganisms contaminating during or post processing, and the intrinsic and extrinsic factors; all of these parameters have an impact on the types and numbers of microorganisms that may be present in minimally processed RTE fruits and vegetables [20]. The counts for the control (CNL) of guacamole at both temperatures (4 or 10 °C) during storage did not exceed 3 log CFU/g, which is likely attributed to (a) the high fat content of the product, (b) the presence of ingredients in the guacamole (also potentially) possessing some degree of antimicrobial function, and (c) the low refrigeration temperatures; all of these limiting or delaying microbial growth. A similar pattern was observed in processed avocados, where initial counts of aerobic mesophilic microorganisms in avocado purees were lower than 3 log CFU/g for all of the treatments [20]. Since RTE salads and other cold meals or snacks are not exposed to heat treatment, they generally have a shorter shelf-life than heated foods.

The antimicrobial effects of citric acid, mastic EO, and chitosan, either individually added and/or as various combinations, were evaluated against both aerobic mesophilic bacteria (TPC; Table 1) and selected species, including LAB (Table 2), *Enterobacteriaceae* (Table 3), yeasts and molds (Table 4), and *Pseudomonas* spp., enumerated in the guacamole samples stored at 4 °C and 10 °C for 7 days.

In the current study, citric acid itself exhibited an antimicrobial effect against the different microbial groups in the guacamole. Similar findings have been reported for sorbic acid (added at 300 mg/kg) to avocado purée, enhancing the microbial stability of the product up to four months [20]. Organic acids, such as citric, sorbic, etc. (being acidic) are able to exert an antimicrobial function, mainly against aerobic microbial species, such as yeasts and molds and *Pseudomonas* spp. [21]. Additionally, inhibition of *Escherichia coli* O157:H7 has been observed in Tabbouleh, a Middle Eastern popular RTE, using a combination of acetic and citric acids [22]. In our study, such action was also evident; however, it can be speculated that the lipid fat content of guacamole could have hindered the effectiveness of the citric acid.

The evolution of aerobic mesophilic bacteria (TPC; Table 1) and selected species, including LAB (Table 2), *Enterobacteriaceae* (Table 3), yeasts and molds (Table 4), and *Pseudomonas* spp., enumerated in the guacamole samples stored at 4 °C and 10 °C for 7 days, both in the absence of treatment (untreated CNL—control) and treated (CA; M; CH; CAM; CACH; CHM and CACHM) is presented (Table 1, Table 2, Table 3, Table 4 and Table 5). The initial TPC of the indigenous microbial load of the guacamole samples (control; CNL samples) was ca. 3.9 and 4.3 log CFU/g at 4 and 10 °C, respectively, under aerobic packaging conditions. Significant (*p* < 0.05) TPC increases in CNL samples, irrespective, of storage temperature, were recorded after day 3 storage, with final (day 7) populations in the same range (2.3–2.4 log CFU/g; Table 1).

At 4 °C, LAB was the dominant species (3.7 log CFU/g; Table 2), closely followed by yeasts and molds (3.6 log CFU/g; Table 4), *Enterobacteriaceae* (Table 3), and *Pseudomonas* spp. (both at ca. 3.3 log CFU/g; Table 5). At 10 °C, the populations of the species examined in our study, by decreasing order, were yeasts and molds (4.6 log CFU/g), *Enterobacteriaceae* (4.5 log CFU/g), LAB (3.9 log CFU/g), and *Pseudomonas* spp. (3.0 log CUF/g).

Of all the species enumerated in the present study, yeasts and molds were the predominant species, followed by LAB, *Enterobacteriaceae*, and *Pseudomonas* spp., at populations ranging from 3.6 to 2.0 and 2.65 to 1.45 (4 and 10 °C, respectively, Table 5, Table 2, Table 3 and Table 4) in the CNL samples, whereas, in general, lower populations (*p* < 0.05) were obtained for all the treated samples and especially those with the two (CACH, CAM, CHM) or triple combinations (CACHM).

**Table 1:** Changes in TPC (log (N_0_/N) ± SD CFU/g) in guacamole in the absence or presence of natural antimicrobials.

Temp	Days	CNL	CA 0.15%	CA 0.3%	M 0.2%	M 0.4%	CH 0.5%	CH 1%	CACH	CAM	CHM	CACHM	*p*-Value
4 °C	0	0.00^fghijklmn^	0.00^fghijklmn^	0.00^fghijklmn^	0.00^fghijklmn^	0.00^fghijklmn^	0.00^fghijklmn^	0.00^fghijklmn^	0.00^fghijklmn^	0.00^fghijklmn^	0.00^fghijklmn^	0.00^fghijklmn^	0.001
	1	0.39^efghijklmn^ ± 0.8	−0.52^lmn^ ± 0.7	0.02^fghijklmn^* ± 0.1	−0.3^jklmn^ ± 0.2	−0.49^lmn^ ± 0.8	−0.31^jklmn^ ± 0.8	−0.46^klmn^* ± 1.7	0.04^fghijklmn^ ± 0.1	0.12^efghijklmn^ ± 0.7	−0.29^jklmn^ ± 0.3	−0.26^jklmn^ ± 0.4	
	3	1.16^abcdefg^ ± 0.3	0.71^cdefghijkl^ ± 1.0	−0.2^ijklmn^* ± 0.2	−0.2^ijklmn^* ± 0.6	−0.14^hijklmn^* ± 0.6	0.43^efghijklmnA^ ± 1.1	−0.41^klmn^* ± 0.5	−0.75^n^* ± 0.6	−0.65^mn^* ± 0.3	0.34^efghijklmn^ ± 0.9	−0.11^ghijklmn^* ± 0.6	
	5	2.32^ab^ ± 0.2	1.06^bcdefghi^ ± 0.3	0.96^cdefghij^ ± 0.4	0.75^cdefghijkl^* ± 0.1	0.8^cdefghijk^* ± 0.3	0.36^efghijklmn^ ± 0.6	0.54^defghijklm^* ± 0.3	0.72^cdefghijkl^* ± 0.4	0.77^cdefghijkl^* ± 0.5	0.19^efghijklmn^* ± 0.6	0.16^efghijklmn^* ± 0.4	
	7	2.36^a^ ± 0.6	1.34^abcde^* ± 0.1	1.71^abc^ ± 0.5	1.58^abcd^* ± 0.1	1.53^abcd^ ± 0.3	1.61^abcd^ ± 0.7	1.23^abcdef^* ± 0.2	1.13^abcdefgh^* ± 0.6	1.4^abcde^* ± 0.3	0.69^cdefghijkl^* ± 0.9	0.49^efghijklmn^* ± 0.5	
10 °C	0	0.00^klmnop^	0.00^klmnop^	0.00^klmnop^	0.00^klmnop^	0.00^klmnop^	0.00^klmnop^	0.00^klmnop^	0.00^klmnop^	0.00^klmnop^	0.00^klmnop^	0.00^klmnop^	0.00
	1	0.29^ijklmno^ ± 0.4	−1.21^p^ ± 0.4	−0.75^op^* ± 0.2	−0.68^op^ ± 0.9	0.2^jklmno^ ± 0.3	−0.03^klmnop^ ± 0.7	0.11^klmnoB^ ± 0.3	−0.31^lmnop^ ± 0.6	0.03^klmno^ ± 0.6	−0.39^mnop^ ± 0.4	−0.5^nop^ ± 0.6	
	3	1.33^abcdefghij^ ± 0.4	0.74^fghijklm^ ± 0.2	1.14^bcdefghijk^* ± 0.2	1.13^bcdefghijk^* ± 0.1	0.79^efghijklm^* ± 0.2	0.91^defghijkl^* ± 0.2	0.66^hijklmn^* ± 0.2	0.95^cdefghijk^* ± 0.2	0.81^efghijklm^* ± 0.4	0.73^fghijklm^ ± 0.3	0.68^ghijklmn^* ± 0.3	
	5	1.99^abcde^ ± 0.9	1.55^abcdefgh^ ± 0.6	1.5^abcdefghi^ ± 0.7	2.2^ab^* ± 0.1	1.61^abcdefgh^* ± 0.5	1.72^abcdefgh^ ± 0.7	1.89^abcdefg^* ± 0.7	1.71^abcdefgh^* ± 0.8	1.89^abcdefg^* ± 0.6	1.8^abcdefgh^* ± 0.6	1.59^abcdefgh^* ± 0.7	
	7	2.34^ab^ ± 0.4	2.11^abcd^* ± 0.4	2.0^abcde^ ± 0.5	2.19^ab^* ± 0.4	2.13^abc^ ± 0.4	2.21^ab^ ± 0.5	1.86^abcdefgh^* ± 0.5	2.41^a^* ± 0.5	1.9^abcdef^* ± 0.5	2.14^abc^* ± 0.4	1.74^abcdefgh^* ± 0.9	
4 °C vs. 10 °C	1	0.798	0.156	0.002	0.383	0.070	0.385	0.000	0.191	0.807	0.637	0.524	
	3	0.498	0.929	0.000	0.007	0.002	0.001	0.023	0.000	0.000	0.274	0.010	
	5	0.359	0.096	0.080	0.000	0.019	0.187	0.019	0.013	0.001	0.000	0.000	
	7	0.938	0.000	0.397	0.005	0.532	0.558	0.000	0.001	0.045	0.002	0.009	

^a, b, c, d, e, f, g, h, i, j, k, l, m, n, o, p, A, B^ Different letters in each treatment indicate significant differences (*p* < 0.05) among the means. * Different letters in each column indicate a significant difference (*p* < 0.05) between the means on the same of storage at 4 and 10 °C. Initial number (N_0_) of TPC in guacamole and bacterial count after storage (N) were 3.89 ± 0.48 and 4.30 ± 0.28 at 4 and 10 °C, respectively. The guacamole (untreated or treated) samples were divided into a total of eleven groups, including untreated CNL—control (absence of antimicrobials) and treated CA—citric acid 0.15%; CA—citric acid 0.3%; M—mastic EO 0.2%; M—mastic EO 0.4%; CH—chitosan 0.5%; CH—chitosan 1%; CAM—citric acid 0.3% + mastic EO 0.4%; CACH—citric acid 0.3% + chitosan 1%; CHM—chitosan 1% + mastic EO 0.4%; and CACHM—citric acid 0.3% + chitosan 1% + mastic EO 0.4%.

**Table 2:** Changes in LAB populations (log (N_0_/N) ± SD CFU/g) in guacamole in the absence or presence of natural antimicrobials.

Temp	Days	CNL	CA 0.15%	CA 0.3%	M 0.2%	M 0.4%	CH 0.5%	CH 1%	CACH	CAM	CHM	CACHM	*p*-Value
4 °C	0	0.00^fghijkl^	0.00^fghijkl^	0.00^fghijkl^	0.00^fghijkl^	0.00^fghijkl^	0.00^fghijkl^	0.00^fghijkl^	0.00^fghijkl^	0.00^fghijkl^	0.00^fghijkl^	0.00^fghijkl^	0.001
	1	0.16^defghijkl^ ± 0.9	0.42^cdefghijkl^* ± 0.3	0.08^efghijkl^* ± 0.1	0.28^defghijkl^ ± 0.1	−0.2^ijkl^* ± 0.4	0.05^efghijkl^ ± 0.0	−0.13^hijkl^ ± 0.4	−0.37^kl^ ± 0.3	−0.26^jkl^ ± 0.3	0.1^efghijkl^ ± 0.1	−0.02^ghijkl^ ± 0.6	
	3	1.08^bcdefg^* ± 0.0	0.94^bcdefgh^ ± 0.2	0.04^efghijkl^* ± 0.1	0.5^cdefghijkl^* ± 0.6	−0.25^jkl^* ± 0.6	−0.54^l^* ± 0.2	−0.34^kl^* ± 0.1	−0.17^hijkl^* ± 0.6	−0.43^kl^ ± 0.3	0.18^defghijkl^ ± 0.4	−0.28^jkl^ ± 0.3	
	5	1.14^bcde^* ± 0.6	0.89^bcdefghi^ ± 0.1	0.26^defghijkl^* ± 0.6	0.82^bcdefghij^* ± 0.3	0.79^bcdefghij^* ± 0.2	−0.04^ghijkl^* ± 0.5	0.61^bcdefghijk^* ± 0.4	0.38^defghijkl^* ± 0.1	0.25^defghijkl^ ± 0.6	0.11^efghijkl^ ± 0.6	0.49^cdefghijkl^ ± 0.2	
	7	2.37^a^ ± 0.1	1.57^ab^ ± 0.5	1.14^bcde^* ± 0.6	1.6^ab^* ± 0.1	1.14^bcde^* ± 0.4	1.11^bcdef^* ± 0.9	0.91^bcdefghi^* ± 0.5	1.53^abc^* ± 0.2	1.25^abcd^ ± 0.2	0.63^bcdefghijk^ ± 0.4	0.84^bcdefghij^ ± 0.4	
10 °C	0	0.00^hi^	0.00^hi^	0.00^hi^	0.00^hi^	0.00^hi^	0.00^hi^	0.00^hi^	0.00^hi^	0.00^hi^	0.00^hi^	0.00^hi^	0.001
	1	0.33^h^ ± 0.8	−1.01^j^* ± 0.1	−0.81^ij^* ± 0.1	−0.27^hij^ ± 0.7	0.37^h^* ± 0.3	0.28^h^ ± 0.3	−0.12^hij^ ± 0.6	−0.21^hij^ ± 0.6	−0.41^hij^ ± 0.7	−0.44^hij^ ± 0.7	−0.27^hij^ ± 0.6	
	3	2.3^abcdefg^* ± 0.2	1.62^g^ ± 1.2	2.27^abcdefg^* ± 0.5	2.24^abcdefg^* ± 0.5	2.05^bcdefg^* ± 0.4	1.94^efg^* ± 0.4	2.17^abcdefg^* ± 0.2	1.98^defg^* ± 0.2	2.17^abcdefg^* ± 0.1	2^cdefg^* ± 0.2	1.83^fg^* ± 0.3	
	5	2.82^abcdef^* ± 0.2	2.37^abcdefg^* ± 0.7	2.82^abcdef^* ± 0.4	2.75^abcdef^* ± 0.2	2.72^abcdef^* ± 0.0	3.07^a^* ± 0.3	2.9^abcde^* ± 0.2	2.77^abcdef^* ± 0.3	2.6^abcdefg^* ± 0.2	2.98^abc^* ± 0.1	2.95^abcde^* ± 0.1	
	7	2.64^abcdef^ ± 0.4	2.82^abcdef^* ± 0.4	2.9^abcde^* ± 0.2	3.06^a^* ± 0.3	2.86^abcde^* ± 0.3	2.9^abcde^* ± 0.3	2.97^abcd^* ± 0.3	3.04^ab^* ± 0.2	2.83^abcdef^* ± 0.4	2.94^abcde^* ± 0.4	2.98^abc^* ± 0.3	
4 °C vs. 10 °C	1	0.765	0.000	0.000	0.234	0.020	0.169	0.976	0.610	0.635	0.097	0.575	
	3	0.000	0.278	0.000	0.001	0.001	0.000	0.000	0.000	0.000	0.000	0.000	
	5	0.000	0.001	0.000	0.000	0.000	0.000	0.000	0.000	0.000	0.000	0.000	
	7	0.173	0.005	0.001	0.000	0.000	0.012	0.001	0.000	0.000	0.000	0.000	

^a, b, c, d, e, f, g, h, i, j, k, l^ Different letters in each treatment indicate significant differences (*p* < 0.05) among the means. * Different letters in each column indicate a significant difference (*p* < 0.05) between the means on the same of storage at 4 and 10 °C. Initial number (N_0_) of TPC in guacamole and bacterial count after storage (N) were 3.89 ± 0.48 and 4.30 ± 0.28 at 4 and 10 °C, respectively. The guacamole (untreated or treated) samples were divided into a total of eleven groups, including untreated CNL—control (absence of antimicrobials) and treated CA—citric acid 0.15%; CA—citric acid 0.3%; M—mastic EO 0.2%; M—mastic EO 0.4%; CH—chitosan 0.5%; CH—chitosan 1%; CAM—citric acid 0.3% + mastic EO 0.4%; CACH—citric acid 0.3% + chitosan 1%; CHM—chitosan 1% + mastic EO 0.4%; and CACHM—citric acid 0.3% + chitosan 1% + mastic EO 0.4%.

**Table 3:** Changes in *Enterobacteriaceae* populations (log (N_0_/N) ± SD CFU/g) in guacamole in the absence or presence of natural antimicrobials.

Temp	Days	CNL	CA 0.15%	CA 0.3%	M 0.2%	M 0.4%	CH 0.5%	CH 1%	CACH	CAM	CHM	CACHM	*p*-Value
4 °C	0	0.00^no^	0.00^no^	0.00^no^	0.00^no^	0.00^no^	0.00^no^	0.00^no^	0.00^no^	0.00^no^	0.00^no^	0.00^no^	0.001
	1	0.00^no^* ± 0.6	0.09^mno^* ± 0.9	0.06^no^* ± 0.2	0.1^lmno^ ± 0.1	−0.36^o^ ± 0.7	−0.11^no^* ± 0.2	0.48^ijklmno^ ± 0.8	−0.06^no^ ± 0.1	0.22^klmno^ ± 0.5	−0.37^o^ ± 0.2	−0.17^no^ ± 0.4	
	3	0.58^hijklmno^ ± 0.6	1.14^defghijklm^* ± 0.4	0.7^ghijklmno^ ± 0.1	0.77^fghijklmn^* ± 0.2	0.36^jklmno^ ± 0.3	0.71^ghijklmno^ ± 0.7	−0.08^no^ ± 0.2	0.09^lmno^* ± 0.2	0.34^jklmno^* ± 0.0	0.03^no^ ± 0.2	0.23^klmno^ ± 0.2	
	5	1.75^abcdefg^ ± 0.9	1.99^abcd^* ± 0.3	1.82^abcdef^ ± 0.7	1.46^bcdefghi^* ± 0.2	1.47^bcdefghi^* ± 0.3	1.59^bcdefgh^* ± 0.1	1.17^cdefghijkl^* ± 0.5	0.9^efghijklmn^* ± 0.1	1.17^defghijklm^* ± 0.5	0.72^ghijklmno^ ± 0.6	0.5^ijklmno^* ± 0.4	
	7	2.77^a^* ± 1.0	2.5^ab^* ± 0.7	2.7^a^* ± 0.3	2.23^abc^ ± 0.6	1.96^abcde^ ± 0.6	1.36^cdefghij^ ± 0.1	1.32^cdefghij^ ± 0.2	1.21^cdefghijk^ ± 0.5	1.49^bcdefghi^ ± 0.1	1.22^cdefghijk^ ± 0.8	0.8^fghijklmn^ ± 0.5	
10 °C	0	0.00^defghij^	0.00^defghij^	0.00^defghij^	0.00^defghij^	0.00^defghij^	0.00^defghij^	0.00^defghij^	0.00^defghij^	0.00^defghij^	0.00^defghij^	0.00^defghij^	0.001
	1	0.53^abcdefghij^* ± 0.2	−0.82^j^* ± 0.2	−0.33^fghij^* ± 0.1	−0.4^ghij^ ± 0.7	−0.82^j^ ± 0.8	−0.55^hij^* ± 0.1	0.01^cdefghij^ ± 0.2	−0.38^fghij^ ± 0.6	−0.81^ij^ ± 0.8	−0.55^hij^ ± 0.8	−0.1^defghij^ ± 0.2	
	3	0.77^abcdefgh^ ± 0.7	0.24^bcdefghij^* ± 0.5	0.52^abcdefghij^ ± 0.4	1.11^abcde^* ± 0.2	0.7^abcdefgh^ ± 0.2	0.91^abcdefg^ ± 0.3	0.6^abcdefghi^ ± 0.8	0.79a^bcdefgh^* ± 0.2	1.25^abcd^* ± 0.3	0.41^abcdefghij^ ± 0.6	0.07^bcdefghij^ ± 0.1	
	5	1.48^ab^ ± 0.6	0.66^abcdefgh^* ± 1.0	1.23^abcde^ ± 0.5	0.76^abcdefgh^* ± 0.1	0.9^abcdefg^* ± 0.1	1.03^abcdef^* ± 0.4	0.65^abcdefgh^* ± 0.4	0.35^abcdefghij^* ± 0.2	0.55^abcdefghij^* ± 0.5	0.54^abcdefghij^ ± 0.9	−0.18^efghijB^ ± 0.5	
	7	1.44^ab^* ± 0.9	0.99^abcdefg^* ± 0.7	0.84^abcdefgh^* ± 0.9	1.42^abc^ ± 0.9	1.46^ab^ ± 0.4	0.86^abcdefgh^ ± 0.9	1.47^ab^ ± 1.2	0.55^abcdefghij^ ± 0.8	1.7^a^ ± 0.9	1.3^abcd^ ± 0.5	0.7^abcdefgh^ ± 0.8	
4 °C vs. 10 °C	1	0.042	0.034	0.023	0.221	0.368	0.000	0.149	0.205	0.075	0.674	0.722	
	3	0.657	0.001	0.420	0.019	0.048	0.456	0.063	0.000	0.003	0.164	0.283	
	5	0.525	0.003	0.151	0.000	0.001	0.017	0.028	0.000	0.025	0.728	0.008	
	7	0.014	0.006	0.023	0.132	0.139	0.228	0.812	0.105	0.665	0.843	0.799	

^a, b, c, d, e, f, g, h, i, j, k, l, m, n, o, B^ Different letters in each treatment indicate significant differences (*p* < 0.05) among the means. * Different letters in each column indicate a significant difference (*p* < 0.05) between the means on the same of storage at 4 and 10 °C. Initial number (N_0_) of TPC in guacamole and bacterial count after storage (N) were 3.89 ± 0.48 and 4.30 ± 0.28 at 4 and 10 °C, respectively. The guacamole (untreated or treated) samples were divided into a total of eleven groups, including: Untreated CNL—control (absence of antimicrobials) and treated CA—citric acid 0.15%; CA—citric acid 0.3%; M—mastic EO 0.2%; M—mastic EO 0.4%; CH—chitosan 0.5%; CH—chitosan 1%; CAM—citric acid 0.3% + mastic EO 0.4%; CACH—citric acid 0.3% + chitosan 1%; CHM—chitosan 1% + mastic EO 0.4%; and CACHM—citric acid 0.3% + chitosan 1% + mastic EO 0.4%.

**Table 4:** Changes in yeasts and molds populations (log (N_0_/N) ± SD CFU/g) in guacamole in the absence or presence of natural antimicrobials.

Temp	Days	CNL	CA 0.15%	CA 0.3%	M 0.2%	M 0.4%	CH 0.5%	CH 1%	CACH	CAM	CHM	CACHM	*p*-Value
4 °C	0	0.00^lmnop^	0.00^lmnop^	0.00^lmnop^	0.00^lmnop^	0.00^lmnop^	0.00^lmnop^	0.00^lmnop^	0.00^lmnop^	0.00^lmnop^	0.00^lmnop^	0.00^lmnop^	0.001
	1	0.11^klmnop^ ± 0.7	−0.27^p^ ± 0.5	−0.2^op^ ± 0.7	1.55^bcdefghi^* ± 0.3	0.31^ijklmnop^ ± 0.2	−0.11^mnop^ ± 0.4	0.45^hijklmnop^ ± 0.4	−0.17^nop^ ± 0.3	0.52^ghijklmnop^* ± 0.3	0.48^ghijklmnop^* ± 0.3	0.19^klmnop^* ± 0.1	
	3	1.93^abcde^ ± 0.6	0.74^ghijklmnop^ ± 0.7	0.24^jklmnop^* ± 0.3	−0.16^nop^* ± 0.3	0.33^ijklmnop^ ± 0.9	0.43^hijklmnop^ ± 0.8	0.16^klmnop^* ± 0.2	0.55^ghijklmnop^ ± 1.0	−0.08^lmnop^* ± 0.5	1.06^efghijklmn^ ± 0.5	0.03^lmnop^ ± 0.6	
	5	2.54^ab^ ± 0.5	1.71^bcdefg^ ± 0.3	1.34^bcdefghijk^* ± 0.7	1.06^fghijklmno^* ± 0.3	1.3^bcdefghijk^ ± 0.5	1.16^cdefghijkl^* ± 0.3	1.05^fghijklmno^* ± 0.5	0.91^ghijklmnop^* ± 0.3	1.05^fghijklmno^* ± 0.2	0.03^lmnop^* ± 0.2	0.62^ghijklmnop^* ± 0.3	
	7	3.11^a^* ± 0.2	1.8^bcdef^ ± 0.0	2.36^abc^ ± 0.6	2.25^abcd^ ± 0.7	2.18^abcd^ ± 0.4	1.45^bcdefghij^ ± 0.1	1.66^bcdefgh^ ± 0.3	1.66^bcdefgh^ ± 0.7	1.95^abcde^ ± 0.1	1.1^defghijklm^* ± 0.9	1.02^fghijklmno^* ± 0.6	
10 °C	0	0.00^jklmno^	0.00^jklmno^	0.00^jklmno^	0.00^jklmno^	0.00^jklmno^	0.00^jklmno^	0.00^jklmno^	0.00^jklmno^	0.00^jklmno^	0.00^jklmno^	0.00^jklmno^	0.00
	1	0.08^jklmno^ ± 0.4	−0.7^no^ ± 0.8	−0.23^lmno^ ± 0.4	−0.23^lmno^* ± 0.4	−0.37^mno^ ± 0.4	0.07^jklmno^ ± 0.8	0.07^jklmno^ ± 0.2	−0.11^klmno^ ± 0.4	0.14^ijklmno^* ± 0.1	−0.98^o^* ± 0.7	−0.66^no^* ± 0.6	
	3	1.61^abcdefgh^ ± 0.5	1.1^defghijk^ ± 0.5	1.62^abcdefgh^* ± 0.2	1.6^abcdefgh^* ± 0.5	0.88^fghijklm^ ± 1.0	1.21^cdefghijk^ ± 0.5	1.06^efghijkl^* ± 0.5	1.06^efghijkl^ ± 0.3	1.26^bcdefghij^* ± 0.6	0.77^ghijklm^ ± 0.4	0.59^hijklmn^ ± 0.6	
	5	2.56^ab^ ± 0.1	1.84^abcdefgh^ ± 0.5	2.11^abcdef^* ± 0.1	2.46^abc^* ± 0.3	1.7^abcdefgh^ ± 0.2	2.4^abcd^* ± 0.4	2.15^abcdef^* ± 0.2	2.16^abcdef^* ± 0.3	2.29^abcde^* ± 0.3	2.14^abcdef^* ± 0.4	1.98^abcdefg^* ± 0.2	
	7	2.66^a^* ± 0.2	2.13^abcdef^ ± 0.4	2.01^abcdefg^ ± 0.5	2.09^abcdefg^ ± 0.4	2.45^abc^ ± 0.0	1.97^abcdefg^ ± 0.5	2.11^abcdef^ ± 0.6	2.13^abcdef^ ± 0.4	1.44^abcdefghi^ ± 0.7	2.38^abcd^* ± 0.2	1.97^abcdefg^* ± 0.5	
4 °C vs. 10 °C	1	0.936	0.389	0.917	0.002	0.049	0.712	0.183	0.762	0.028	0.002	0.019	
	3	0.483	0.390	0.001	0.002	0.386	0.046	0.014	0.230	0.029	0.302	0.137	
	5	0.924	0.672	0.036	0.003	0.107	0.013	0.001	0.000	0.000	0.001	0.000	
	7	0.032	0.109	0.398	0.756	0.190	0.061	0.294	0.160	0.108	0.005	0.005	

^a, b, c, d, e, f, g, h, i, j, k, l, m, n, o, p^ Different letters in each treatment indicate significant differences (*p* < 0.05) among the means. * Different letters in each column indicate a significant difference (*p* < 0.05) between the means on the same of storage at 4 and 10 °C. Initial number (N_0_) of TPC in guacamole and bacterial count after storage (N) were 3.89 ± 0.48 and 4.30 ± 0.28 at 4 and 10 °C, respectively. The guacamole (untreated or treated) samples were divided into a total of eleven groups, including: Untreated CNL—control (absence of antimicrobials) and treated CA—citric acid 0.15%; CA—citric acid 0.3%; M—mastic EO 0.2%; M—mastic EO 0.4%; CH—chitosan 0.5%; CH—chitosan 1%; CAM—citric acid 0.3% + mastic EO 0.4%; CACH—citric acid 0.3% + chitosan 1%; CHM—chitosan 1% + mastic EO 0.4%; and CACHM—citric acid 0.3% + chitosan 1% + mastic EO 0.4%.

**Table 5:** Changes in *Pseudomonas* spp. populations (log (N_0_/N) ± SD CFU/g) in guacamole in the absence or presence of natural antimicrobials.

Temp	Days	CNL	CA 0.15%	CA 0.3%	M 0.2%	M 0.4%	CH 0.5%	CH 1%	CACH	CAM	CHM	CACHM	*p*-Value
4 °C	0	0.00^nopqr^	0.00^nopqr^	0.00^nopqr^	0.00^nopqr^	0.00^nopqr^	0.00^nopqr^	0.00^nopqr^	0.00^nopqr^	0.00^nopqr^	0.00^nopqr^	0.00^nopqr^	0.001
	1	0.15^lmnopqr^ ± 0.4	−0.46^opqr^ ± 0.2	−0.47^pqr^ ± 0.5	0.74^efghijklmn^ ± 0.5	0.09^lmnopqr^ ± 0.1	−0.6^qr^ ± 0.5	0.03^nopqr^ ± 0.7	−0.47^pqr^ ± 0.5	0.28^ijklmnopqr^ ± 0.8	−0.72^r^ ± 0.4	−0.67^qr^ ± 0.5	
	3	0.33^ijklmnopqr^ ± 0.3	0.32^ijklmnopqr^ ± 0.2	0.84^efghijklmn^ ± 0.3	0.57^ghijklmnop^ ± 0.6	0.2^jklmnopqr^ ± 0.1	0.25^ijklmnopqr^ ± 0.2	0.2^jklmnopqr^ ± 0.4	0.21^jklmnopqr^ ± 0.1	0.06^mnopqr^ ± 0.5	−0.11^nopqr^ ± 0.8	0.45^hijklmnopq^ ± 0.6	
	5	1.95^abc^ ± 0.0	1.46^abcdefg^ ± 0.6	1.37^abcdefghi^ ± 0.4	1.02^efghijklmn^ ± 0.4	2.18^a^ ± 0.1	1.31^bcdefghijk^ ± 0.3	1.19^defghijklm^ ± 0.5	1.23^cdefghijkl^ ± 0.1	1.0^efghijklmn^ ± 0.4	0.18^klmnopqr^ ± 0.3	0.69^fghijklmno^ ± 0.8	
	7	1.97^abc^ ± 0.5	2.02^ab^ ± 0.1	1.8^abcd^ ± 0.0	1.42^abcdefgh^ ± 0.1	2.22^a^ ± 0.5	1.58^abcdef^ ± 0.2	1.35^abcdefghij^ ± 0.2	1.69^abcde^ ± 0.6	1.83^abcd^ ± 0.3	1.23^cdefghijkl^ ± 0.8	0.99^efghijklmn^ ± 0.5	
10 °C	0	0.00^k^	0.00^k^	0.00^k^	0.00^k^	0.00^k^	0.00^k^	0.00^k^	0.00^k^	0.00^k^	0.00^k^	0.00^k^	0.001
	1	0.71^efghijk^ ± 0.2	0.62^ghijk^ ± 0.3	0.39^ijk^ ± 0.5	0.77^defghijk^ ± 0.2	0.64^fghijk^ ± 0.4	0.47^hijk^ ± 0.6	0.48^hijk^ ± 0.3	0.39^ijk^ ± 0.5	0.52^ghijk^ ± 0.3	0.76^defghijk^ ± 0.3	0.36^jk^ ± 0.3	
	3	1.4^abcdefghij^ ± 0.2	1.43^abcdefghij^ ± 0.3	1.51^abcdefghij^ ± 0.3	1.24^bcdefghijk^ ± 0.2	1.45^abcdefghij^ ± 0.6	1.42^abcdefghij^ ± 0.5	0.73^efghijk^ ± 0.5	1.14^bcdefghijk^ ± 0.4	1.7^abcdefghi^ ± 0.3	1.03^bcdefghijk^ ± 0.8	0.82^defghijk^ ± 0.5	
	5	2.19^ab^ ± 0.6	1.62^abcdefghij^ ± 0.3	1.34^bcdefghijk^ ± 0.8	1.27^bcdefghijk^ ± 1.0	2.07^abcd^ ± 0.2	1.5^abcdefghij^ ± 1.2	0.71^efghijk^ ± 0.4	1.82^abcdefg^ ± 0.3	1.76^abcdefgh^ ± 0.4	1.48^abcdefghij^ ± 0.6	0.85^cdefghijk^ ± 0.5	
	7	2.4^a^ ± 0.1	1.73^abcdefghi^ ± 0.5	1.72^abcdefghi^ ± 0.1	1.25^bcdefghijk^ ± 0.7	1.99^abcde^ ± 0.3	1.78^abcdefgh^ ± 0.7	1.53^abcdefghij^ ± 0.7	1.82^abcdefg^ ± 0.6	2.17^abc^ ± 0.1	1.98^abcdef^ ± 1.0	0.89^bcdefghijk^ ± 0.9	
4 °C vs. 10 °C	1	0.075	0.003	0.005	0.937	0.013	0.004	0.366	0.005	0.457	0.000	0.000	
	3	0.000	0.000	0.031	0.099	0.006	0.006	0.051	0.000	0.000	0.020	0.234	
	5	0.428	0.603	0.952	0.582	0.314	0.692	0.108	0.007	0.005	0.001	0.714	
	7	0.103	0.347	0.230	0.669	0.431	0.555	0.607	0.727	0.025	0.212	0.780	

^a, b, c, d, e, f, g, h, i, j, k, l, m, n, o, p, q, r^ Different letters in each treatment indicate significant differences (*p* < 0.05) among the means. Initial number (N_0_) of TPC in guacamole and bacterial count after storage (N) were 3.89 ± 0.48 and 4.30 ± 0.28 at 4 and 10 °C, respectively. The guacamole (untreated or treated) samples were divided into a total of eleven groups, including: Untreated CNL—control (absence of antimicrobials) and treated CA—citric acid 0.15%; CA—citric acid 0.3%; M—mastic EO 0.2%; M—mastic EO 0.4%; CH—chitosan 0.5%; CH—chitosan 1%; CAM—citric acid 0.3% + mastic EO 0.4%; CACH—citric acid 0.3% + chitosan 1%; CHM—chitosan 1% + mastic EO 0.4%; and CACHM—citric acid 0.3% + chitosan 1% + mastic EO 0.4%.

Regarding mastic EO, only moderate inhibitory effects were observed when added singly to the salad, whereas in combination with chitosan, a more effective treatment was noted. In other studies, and also depending on the product matrix, mixed results have been demonstrated [23,24]. Additionally, previous studies involving EOs including oregano, thyme, and rosemary, have shown to delay the microbial growth of RTE vegetables, even at concentrations below their minimum inhibitory concentration (MIC) values [25]. The antimicrobial activity of EOs (largely due to their phenolic constituents) has been attributed to their ability to disrupt cell membranes, reduce ATP generation, inhibit protein synthesis, and cause pH imbalance [10].

On day 7, reductions (especially under CACHM treatment) were more prominent at 4, compared to 10 °C, and as previously noted for the yeasts, and molds, LAB, *Enterobacteriaceae*, and *Pseudomonas* spp. (Table 2, Table 3, Table 4 and Table 5). Reductions in the range 1 to 2 log CFU/g were obtained for *Pseudomonas* spp., *Enterobacteriaceae*, and yeasts, and molds under CACHM treatment, whereas interestingly, for LAB the highest reduction (1.74 log CFU/g) was achieved by CHM, followed by CACHM (1.5 log CFU/g). This decreasing trend was also noted at 10 ^o^C, except for the LAB species. Regarding, the combinations of two antimicrobials, the presence of chitosan (CACH) especially at the low storage temperature (4 ^o^C) was also effective, in most cases, resulting in substantial decreases of the microbial populations examined. At 10 ^o^C, of all the treatments, CACHM was, in general, most effective, followed by CACH, CAM and CHM combinations, with reductions recorded for all microbial species examined, compared to the control samples (CNL).

Chitosan, arguably, was the most effective of all the antimicrobials tested in our study, added either singly or combined. The role of chitosan and its effects on the quality and safety of RTE foods have been recently summarized [26]. The antimicrobial effect of chitosan depends heavily on the concentration used [27]. Chitosan coatings containing EOs have been reported to extend the shelf-life of fresh produce, such as blueberries [28]. Chitosan is a promising natural agent for improving food safety through antimicrobial action, packaging, and sanitation. Its multifunctional capabilities align with the modern demand for clean-label and sustainable food preservation methods. Continued research and development are essential to harness its full potential. Despite its promising applications, challenges such as solubility issues and the potential for allergenicity remain. Future research should focus on improving chitosan formulations and understanding its interactions within complex food matrices [29,30], and that also includes avocado-based product formulations. For food manufacturers, the challenge is twofold: safeguarding refrigerated foods from real risks while also reassuring consumers that the quality and the taste of their favorite offerings remain uncompromised.

Finally, what needs to be carefully evaluated is the evolution of the microbiota of the guacamole, by itself or under various antimicrobial or added preservatives, and especially the role of the lipid content of the avocado-based salad product formulations (guacamole, salsa, etc.) on the microbial species, chemical oxidation parameters, etc. Various fermentation-based ingredients such as cultured dextrose, vinegar, cultured sugar, and nature-based ferments, unlike traditional preservatives such as sorbates or benzoates, can be intergrated into sala formulations without affecting taste or texture, which is crucial for categories where flavor authenticity is paramount, such as RTE deli saladds, soups or sauces. Such novel antimicrobials enable manufacturers to reduce waste by extending freshness windows and overall shelf-life [26].

## 4. Conclusions

The present study demonstrated primarily that of all the species enumerated, yeasts and molds were the predominant species, followed by Lactic Acid Bacteria (LAB), *Enterobacteriaceae*, and *Pseudomonas* spp., in guacamole. No clear synergistic effects were observed among the natural antimicrobials. Refrigeration acted as an important additional barrier, effectively slowing microbial growth and keeping low counts in the avocado-based salad (absence of antimicrobials) over the 7-day storage period. Overall, the application of citric acid, alone or in combination with mastic oil or chitosan, together with refrigeration, can effectively help maintain the microbial safety and quality of RTE avocado-based salad products (such as guacamole, salsa, etc.). Future studies are needed to assess the microbiological and sensorial shelf-life of guacamole. Such studies are important to verify the acceptability of RTE avocado-based salad products. Most importantly to safeguard consumers and reduced hazards, the role of emerging technologies, such as modified atmosphere or vacuum packaging (MAP), must also be considered.

## Data Availability

The original contributions presented in the study are included in the article; further inquiries can be directed to the corresponding author.
